# Laboratory variables as predictors of progression in gastroenteropancreatic neuroendocrine tumors in different lines of antineoplastic treatments

**DOI:** 10.31744/einstein_journal/2022AO6985

**Published:** 2022-05-24

**Authors:** Daniela Pezzutti Domigues Armentano, Mariana Ribeiro Monteiro, Pedro Nazareth Aguiar, Jessica Sayuri Tsukamoto, Raquel Baptista Pio, Renata Arakelian, Raphael Leonardo Cunha Araujo, Pedro Luiz Serrano Usón

**Affiliations:** 1 Americas Oncology Group São Paulo SP Brazil Americas Oncology Group, São Paulo, SP, Brazil.; 2 Hospital Israelita Albert Einstein São Paulo SP Brazil Hospital Israelita Albert Einstein, São Paulo, SP, Brazil.

**Keywords:** Biomarkers, Chromogranin A, Neuroendocrine tumors, Gastro-enteropancreatic neuroendocrine tumor, Blood platelets, Neutrophils, Lutetium, Intention to treat analysis

## Abstract

**Objective:**

To determine the association of red cell blood counts, and liver panel tests to predict outcomes in patients with gastroenteropancreatic neuroendocrine tumors who underwent systemic antineoplastic treatments.

**Methods:**

Patients with gastroenteropancreatic neuroendocrine tumors in systemic treatment were assessed according to laboratory tests within the same period. Progression free survival was determined by the period between the beginning of treatment and the date of progression. We used conditional models (PWP model) to verify the association between laboratory tests and tumor progression. The level of significance used was 5%.

**Results:**

A total of 30 treatments given to 17 patients in the intention-to-treat population were evaluated. Treatment included octreotide, lanreotide, everolimus, lutetium, and chemotherapy. We had statistically significant results in chromogranin A, neutrophils and platelets-to-lymphocyte ratio. The risk of progression increases by 2% with the addition of 100ng/mL of chromogranin A (p=0.034), 4% with the increase of 100 neutrophil units (p=0.006), and 21% with the addition of 10 units in platelets-to-lymphocyte ratio (p=0.002).

**Conclusion:**

Chromogranin A, neutrophils and platelets-to-lymphocyte ratio were associated with disease progression during systemic treatment in gastroenteropancreatic neuroendocrine tumors. Further prospective studies with larger cohorts are necessary to validate our findings.

## INTRODUCTION

Neuroendocrine neoplasms account for only 0.5% of diagnosed neoplasia cases,^(1)^ which is a rare disease with an average incidence of 2/100,000 and that around 60% of cases originate from the digestive system.^(2–4)^ Most gastroenteropancreatic neuroendocrine tumors (GEP-NET) diagnosed are asymptomatic,^(2)^ and treatment in case of advanced disease includes several options that range from somatostatin analogues, tyrosine kinase inhibitors, radioisotopes such as lutetium 177, and even chemotherapy.^(5,6)^

Part of the difficulty in choosing the systemic treatment rises from the lack of validated indicators to evaluate the response to multiple available treatments. Image workout as computed tomography has difficulty to conclude progression due to their indolent behavior.

Chromogranin A marker used for diagnosis presents conflicting results in the published literature regarding the actual ability to monitor treatment response.^(7,8)^ We still lack validated indicators to evaluate the response in GEP-NET to the multiple treatments available. Moreover, other laboratory tests that usually present association with other tumors of the digestive tract have been poorly studied in neuroendocrine tumors. In addition, there is no current recommendation about evaluating laboratory tests in GEP-NET.^(9,10)^

## OBJECTIVE

To determine the association of red cell blood counts, and liver panel tests to predict outcomes in patients with gastroenteropancreatic neuroendocrine tumors who underwent systemic antineoplastic treatments.

## METHODS

### Patients

We included patients with GEP-NET who were admitted to the *Hospital Israelita Albert Einstein* (HIAE) and Americas Oncology Group in São Paulo, Brazil, from January 2006 to December 2018. Patients with incomplete data were excluded from the analysis of the study. Data collected included patient’s sex and age, clinical stage, and if available, the pathologic stage at diagnosis, location of the primary tumor, location of metastasis, hepatic tumor volume, tumor grade, Ki-67, previous treatments, surgery of the primary tumor, metastasectomy, trans-arterial, and radiotherapy treatments.

Each treatment was assessed in the same period individually based on the laboratory findings such as gamma-glutamyltransferase (GGT), alkaline phosphatase (AP), chromogranin A (CgA), neutrophils, lymphocyte and platelets, neutrophil-to-lymphocyte ratio (NLR), and platelets-to-lymphocyte ratio (PLR). Neutrophil-to-lymphocyte ratio higher than 3 and a PLR higher than 150 were considered as cutoff points for analysis that were also associated with worse oncologic outcomes in gastrointestinal cancers.^(9,10)^

Time to progression was determined by the period between the beginning of treatment to be evaluated, and the date of progression. The study was approved by the research and ethical committees of both institutions in accordance with the existing national standards (CAAE: 81744017.6.0000.0071, protocol: 2.489.784, and CAAE: 64273317.9.0000.5533, protocol: 4.324.677). The consent term was waived since this was a retrospective study. All datasets in which the conclusions of this report were based on are available upon request.

### Statistical analysis

The quantitative variables were described using median and interquartile range (IIQ: 1^st^ and 3^rd^ quartiles), and the qualitative variables were described by means of absolute and relative frequencies.^(11)^ The association between laboratory tests and tumor progression were evaluated by conditional models developed by Prentice, Williams and Peterson (PWP model).^(12)^ Such survival models deal with recurrent events such as repetition of the occurrence of progression and time-dependent variables such as the tests that were collected at more than one time and the status of progression.^(13)^ The statistical package R was used for the analyzes.^(14)^ The level of significance adopted was 5%.

## RESULTS

Thirty treatments were provided to 17 patients as an intention-to-treat population (median 2 treatment per patient). Table 1 presents the description of the demographic variables, staging, and locations of the primary tumor. The median age of patients was 58 years (ranging 26-91). The majority of them were women (∼65%), and ∼30% of individuals had hepatic tumor volume >50%, ∼90% metastatic cancer at the time of diagnosis, and ∼17% (n=3) died because of the disease progression.

**Table 1:** Clinical and pathological characteristics of the patients who underwent systemic treatment of gastroenteropancreatic neuroendocrine tumors

Variable		Total	Progression	
			No n (%)	Yes n (%)
Sex - (n=17), n (%)				
	Male	6 (35.3)	2 (33.3)	4 (36.4)
	Female	11 (64.7)	4 (66.7)	7 (63.6)
Location - (n=17), n (%)				
	Pancreatic	6 (35.3)	2 (33.3)	4 (36.4)
	Ileum	5 (29.4)	2 (33.3)	3 (27.3)
	Small intestine	6 (35.3)	2 (33.3)	4 (36.4)
Hepatic tumor volume - (n=14), n (%)				
	<25%	4 (28.6)	3 (75.0)	1 (10.0)
	>=25% and <=50%	6 (42.9)	1 (25.0)	5 (50.0)
	> 50%	4 (28.6)	0 (0.0)	4 (40.0)
Ki-67 (%)				
	Median [IQR]	7.70 [2.75-10.00]	6.50 [2.25-10.00]	7.70 [5.00-10.00]
	Minimum-Maximum (n)	1.00-35.00 (16)	1.00-15.00 (6)	1.00-35.00 (10)
Stage at diagnosis - (n=17), n (%)				
	Metastatic	15 (88.2)	4 (66.7)	11 (100.0)
	Non-Metastatic	2 (11.8)	2 (33.3)	0 (0.0)
Stage (T) at diagnosis - (n=17), n (%)				
	Unknown	11 (64.7)	2 (33.3)	9 (81.8)
	T2	2 (11.8)	0 (0.0)	2 (18.2)
	T3	3 (17.6)	3 (50.0)	0 (0.0)
	T4	1 (5.9)	1 (16.7)	0 (0.0)
Stage (N) at diagnosis - (n=17), n (%)				
	N0	2 (11.8)	2 (33.3)	0 (0.0)
	N1	5 (29.4)	2 (33.3)	3 (27.3)
	Unknown	10 (58.8)	2 (33.3)	8 (72.7)
Surgery - (n=17), n (%)				
	No	7 (41.2)	2 (33.3)	5 (45.5)
	Yes	10 (58.8)	4 (66.7)	6 (54.5)
Local therapy - (n=17), n (%)				
	No	10 (58.8)	4 (66.7)	6 (54.5)
	Yes	7 (41.2)	2 (33.3)	5 (45.5)

IQR: interquartile range.

The metastasis locations, previous treatments, and systemic therapies are demonstrated in table 2, and the results of laboratory tests in appendix 1. Around 80% of patients had liver metastasis, one-third were treated with octreotide, and about 25% (n=8) were treated with lutetium.

**Table 2:** Characteristics and therapies to individuals’ lines of treatment

Variable		Total	Progression	
			No (n=12)	Yes (n=18)
Metastasis location, n (%)				
	Liver	25 (83.3)	10 (83.3)	15 (83.3)
	Peritoneum	4 (13.3)	2 (16.7)	2 (11.1)
	Bone	1 (3.3)	0 (0.0)	1 (5.6)
Grade, n (%)				
	1	9 (30.0)	3 (25.0)	6 (33.3)
	2	18 (60.0)	7 (58.3)	11 (61.1)
	3	3 (10.0)	2 (16.7)	1 (5.6)
Metastasectomy, n (%)				
	No	25 (83.3)	11 (91.7)	14 (77.8)
	Yes	5 (16.7)	1 (8.3)	4 (22.2)
Treatment, n (%)				
	Octreotide LAR30mg	9 (30.0)	1 (8.3)	8 (44.4)
	Octreotide LAR60mg	1 (3.3)	1 (8.3)	0 (0.0)
	Lanreotide 120mg	5 (16.7)	1 (8.3)	4 (22.2)
	Everolimus	4 (13.3)	1 (8.3)	3 (16.7)
	Lutetium	8 (26.7)	6 (50.0)	2 (11.1)
	Chemotherapy	3 (10.0)	2 (16.7)	1 (5.6)
Line of treatment, n (%)				
	First	13 (43.3)	4 (33.3)	9 (50.0)
	Second	9 (30.0)	5 (41.7)	4 (22.2)
	Third	4 (13.3)	2 (16.7)	2 (11.1)
	Fourth	3 (10.0)	0 (0.0)	3 (16.7)
	Fifth	1 (3.3)	1 (8.3)	0 (0.0)
Progression or last follow-up time (days)				
	Median [IQR]	268.50 [146.00-546.50]	456.00 [228.75-1,348.00]	187.50 [93.25-350.50]

LAR: long-acting release; IQR: interquartile range.

The results of the models for the risk of occurrence of progression given the results of the laboratory tests over time are presented in table 3. All factors collected for risk of progression had risk ratio >1, except lymphocytes (risk ratio <1). Statistically meaningful results were observed in CgA, neutrophils, and PLR. The risk of progression increases by 2% with the addition of 100ng/mL of CgA (p=0.034), the risk of progression increases by 4% with the increase of 100 neutrophil units (p=0.006), and the risk of progression increased by 21% with the adding of 10 units in PLR (p=0.002).

**Table 3:** Prentice-Williams-Peterson models for progression and laboratory tests as explanatory variables

Variable	Sample size	Odds ratio (95%CI)	p value
Chromogranin A (ng/mL)†	45	1.02 (1.00-1.05)	0.034
(GGT-U/L)*	67	1.04 (0.99-1.09)	0.117
(AP-U/L)*	71	1.05 (0.99-1.11)	0.098
Neutrophils†	62	1.04 (1.01-1.07)	0.006
Lymphocytes†	60	0.96 (0.87-1.06)	0.398
Platelet‡	72	1.01 (0.99-1.02)	0.414
NLR	60	1.02 (0.92-1.12)	0.752
PLR*	60	1.21 (1.07-1.37)	0.002

* consider the addition of 10 for interpretation;

† consider the addition of 100 for interpretation and

‡ consider the addition of 1000 for interpretation.

95%CI: 95% confidence interval; GGT: gamma-glutamyltransferase; AP: alkaline phosphatase; NLR: neutrophil-to-lymphocyte ratio; PLR: platelet-to-lymphocyte ratio.

## DISCUSSION

The use of CgA has been extensively studied in the last years. These studies have shown that patients with high levels of CgA have worst prognosis.^(15–17)^ In addition, preoperative CgA levels has also been related to recurrence of disease following resection of the primary tumor.^(18)^ Regarding the ability to predict progression during treatment in advanced disease, the results of studies published in the literature so far present conflicting data, with groups demonstrating positive association of the marker with progression,^(19,20)^ and others demonstrating poor correlation.^(21,22)^

Concerning patients with GEP-NET, CgA, neutrophils and PLR were correlated with disease progression. These three biomarkers were progressively related. The higher the value the greater the risk of progression. We noticed a tendency to control disease and high levels of lymphocytes, however, without statistical significance. In our study, the risk of progression increased by 2% with each addition of 100ng/mL of CgA (p value=0.034). We must emphasize the limitations of this marker that can directly influence different results found in the literature including, *e.g*., the lack of assay standardization, and generation of significant variations across different laboratories. Other conditions that can affect CgA levels should be considered such as gastrointestinal and cardiovascular disorders and ultimately, some GEP-NET (30-50%) which do not show elevated CgA levels.^(15,23,24)^

It is known that high neutrophil counts contribute to the survival of tumor cells due to the inhibition of lymphoid cells.^(25)^ Tumor-associated neutrophils and neutrophils in the bloodstream of patients with advanced cancer are associated with poor prognosis in several tumors.^(26–28)^ One study evaluated patients with neuroendocrine tumors (NET), including GEP-NET, and found that elevated neutrophil counts have association with worse overall survival.^(29)^ In our study, we demonstrated the importance of neutrophil levels in the patient’s bloodstream, with the risk of progression increasing by 4% with the increase of 100 neutrophil units (p=0.006) during systemic treatment. Neutrophil-to-lymphocyte ratio was associated in other studies with worse outcomes in GEP-NET.^(30)^

An association was not found in this study despite the risk associated with neutrophils at high levels and the apparent protective effect of lymphocytes for progression, although with no statistical significance. We believe that this absence of association between NLR and progression-free survival is probably because of an adequate cutoff point that has not been well defined yet for NLR, given that studies have used several cutoff points ranging from 2-5.^(10,30–32)^

It seems that platelets also contribute to metastatic mechanisms by protecting circulating tumor cells.^(33)^ In agreement with these evidence, PLR seems to be able to estimate the magnitude of systemic inflammation in cancer patients.^(10,34)^ A meta-analysis with 17 cohorts defined that the PLR ratio is a potential marker in pancreatic adenocarcinoma.^(35)^ Based on our results, each addition of 10 units beyond the cutoff point 150 in PLR increases the risk of progression by 21% (p=0.002). These association were also observed in pancreatic NET by another group who demonstrated that PLR is an independent predictor of disease progression.^(36)^

There were some limitations in our study. First, our data are limited by the retrospective nature of data analyses. Second, this study was based in a relatively small sample of patients. Third, around 70% of the patients were treated with somatostatin analogs and peptide receptor radionuclide therapy. For this reason, in earlier lines of systemic treatment, therefore, the results of this study should be taken with caution in other settings. However, despite these limitations, our analysis is consistent with previous findings reported by other research groups.^(19,29,30,36,37)^

## CONCLUSION

The laboratory variables chromogranin A, neutrophils, and platelets-to-lymphocyte ratio are associated with disease progression during systemic treatment for gastroenteropancreatic neuroendocrine tumors. Prospective initiatives using these factors could demonstrate the real impact of these biomarkers on different treatment strategies.

## Appendix 1 Laboratory tests and values in different evaluated periods

**Table:**

Variable		Total	Progression	
			No	Yes
Baseline chromogranin A (CgA) (ng/mL)				
	Median [IQR]	316.50 [25.50-640.00]	382.00 [138.00-630.00]	312.00 [25.00-535.00]
	Minimum-Maximum (n)	2.70-11,060.00 (20)	2.70-8,531.00 (9)	4.00-11,060.00 (11)
CgA (1-3 months)				
	Median [IQR]	206.00 [52.22-538.50]	90.00 [48.55-458.00]	259.00 [153.00-690.00]
	Minimum-Maximum (n)	2.00-6,335.00 (12)	17.00-3,159.00 (7)	2.00-6,335.00 (5)
CgA (3-6 months)				
	Median [IQR]	139.00 [9.40-493.00]	93.10 [11.00-468.00]	325.50 [40.60-5,710.25]
	Minimum-Maximum (n)	3.20-8,531.00 (11)	3.20-474.00 (5)	6.40-8,531.00 (6)
CgA (6-12 months)				
	Median [IQR]	87.00 [67.00-106.00]	114.00 [106.00-122.00]	58.00 [49.00-67.00]
	Minimum-Maximum (n)	40.00-130.00 (4)	98.00-130.00 (2)	40.00-76.00 (2)
Baseline GGT (U/L)				
	Median [IQR]	72.50 [31.50-217.25]	74.00 [28.50-211.50]	71.00 [40.00-208.50]
	Minimum-Maximum (n)	15.00-518.00 (26)	15.00-435.00 (11)	21.00-518.00 (15)
GGT (1-3 months)				
	Median [IQR]	47.00 [28.00-208.00]	36.50 [23.50-169.50]	74.00 [29.00-411.00]
	Minimum-Maximum (n)	13.00-435.00 (25)	13.00-359.00 (12)	16.00-435.00 (13)
GGT (3-6 months)				
	Median [IQR]	189.00 [66.25-284.00]	123.50 [44.75-198.50]	292.00 [131.25-360.50]
	Minimum-Maximum (n)	22.00-457.00 (12)	29.00-273.00 (6)	22.00-457.00 (6)
GGT (6-12 months)				
	Median [IQR]	75.00 [28.00-221.50]	148.50 [91.25-205.75]	75.00 [22.00-180.00]
	Minimum-Maximum (n)	17.00-297.00 (7)	34.00-263.00 (2)	17.00-297.00 (5)
Baseline alkaline phosphatase (AP-U/L)				
	Median [IQR]	125.00 [99.50-161.00]	129.00 [91.50-159.00]	124.00 [105.50-161.00]
	Minimum-Maximum (n)	50.00-431.00 (27)	50.00-378.00 (11)	65.00-431.00 (16)
AP (1-3 months)				
	Median [IQR]	125.50 [92.00-160.25]	120.00 [86.50-131.00]	129.00 [96.00-190.00]
	Minimum-Maximum (n)	53.00-667.00 (26)	53.00-161.00 (11)	76.00-667.00 (15)
AP (3-6 months)				
	Median [IQR]	144.50 [116.75-171.00]	144.00 [105.50-145.00]	176.00 [119.50-254.50]
	Minimum-Maximum (n)	91.00-614.00 (14)	91.00-156.00 (7)	99.00-614.00 (7)
AP (6-12 months)				
	Median [IQR]	135.00 [93.50-172.00]	177.50 [156.25-198.75]	95.00 [92.00-161.00]
	Minimum-Maximum (n)	83.00-220.00 (7)	135.00-220.00 (2)	83.00-183.00 (5)
Baseline neutrophils				
	Median [IQR]	4,110.50 [2,376.25-4,965.25]	3,447.00 [2,348.75-4,120.00]	4,479.00 [2,769.25-5,656.50]
	Minimum-Maximum (n)	929.00-10,331.00 (24)	1,749.00-4,283.00 (8)	929.00-10,331.00 (16)
Neutrophils (1-3 months)				
	Median [IQR]	3,534.50 [2,242.00-4,075.00]	2,740.00 [2,168.25-3,434.75]	3,848.00 [2,386.75-4,268.00]
	Minimum-Maximum (n)	1,520.00-11,294.00 (22)	1,520.00-5,026.00 (8)	1,650.00-11,294.00 (14)
Neutrophils (3-6 months)				
	Median [IQR]	3,807.00 [2,813.00-4,773.50]	3,267.00 [2,881.25-4,493.50]	4,119.00 [2,431.25-4,621.00]
	Minimum-Maximum (n)	929.00-5,859.00 (12)	2,693.00-5,007.00 (6)	929.00-5,859.00 (6)
Neutrophils (6-12 months)				
	Median [IQR]	3,564.00 [2,700.00-3,928.00]	2,700.00 [2,700.00-2,700.00]	3,746.00 [3,285.50-4,462.00]
	Minimum-Maximum (n)	2,450.00-6,064.00 (5)	2,700.00-2,700.00 (1)	2,450.00-6,064.00 (4)
Baseline lymphocytes				
	Median [IQR]	1,811.00 [1,147.00-2,088.50]	2,088.50 [1,732.75-2,339.75]	1,708.00 [996.50-1,885.50]
	Minimum-Maximum (n)	579.00-2,880.00 (23)	601.00-2,880.00 (8)	579.00-2,868.00 (15)
Lymphocytes (1-3 months)				
	Median [IQR]	1,778.00 [1,187.00-2,049.00]	1,526.50 [1,108.50-1,823.75]	1,903.00 [1,187.00-2,102.00]
	Minimum-Maximum (n)	519.00-2,726.00 (21)	519.00-2,050.00 (8)	583.00-2,726.00 (13)
Lymphocytes (3-6 months)				
	Median [IQR]	1,820.50 [940.25-2,083.00]	1,531.00 [993.50-2,023.50]	1,840.50 [1,140.50-2,459.50]
	Minimum-Maximum (n)	579.00-2,751.00 (12)	711.00-2,140.00 (6)	579.00-2,751.00 (6)
Lymphocytes (6-12 months)				
	Median [IQR]	1,662.00 [1,195.00-2,114.25]	1,241.00 [1,241.00-1,241.00]	2,083.00 [1,570.00-2,145.50]
	Minimum-Maximum (n)	1,057.00-2,208.00 (4)	1,241.00-1,241.00 (1)	1,057.00-2,208.00 (3)
Baseline platelets				
	Median [IQR]	225,000 [196,500-330,500]	219,000 [200,500-300,000]	233,500 [193,000-362,000]
	Minimum-Maximum (n)	109,000-560,000 (27)	109,000-354,000 (11)	140,000-560,000 (16)
Platelets (1-3 months)				
	Median [IQR]	209,000 [168,000-317,000]	197,000 [154,500-270,000]	274,500 [185,250-325,500]
	Minimum-Maximum (n)	130,000-542,000 (25)	130,000-349,000 (11)	133,000-542,000 (14)
Platelets (3-6 months)				
	Median [IQR]	216,500 [173,000-259,500]	210,000 [167,000-248,000]	225,000 [184,000-255,000]
	Minimum-Maximum (n)	142,000-391,000 (16)	142,000-391,000 (7)	146,000-341,000 (9)
Platelets (6-12 months)				
	Median [IQR]	162,000 [149,500-186,500]	170,000 [166,000-174,000]	156,000 [143,000-195,000]
	Minimum-Maximum (n)	54,000-305,000 (7)	162,000-178,000 (2)	54,000-305,000 (5)
NLR (baseline)				
	Median [IQR]	2.18 [1.21-3.42]	1.72 [1.09-2.45]	2.90 [1.42-3.88]
	Minimum-Maximum (n)	0.66-13.12 (23)	0.66-5.06 (8)	0.80-13.12 (15)
NLR (1-3 months)				
	Median [IQR]	1.98 [1.23-2.96]	2.20 [1.56-2.68]	1.98 [1.23-3.13]
	Minimum-Maximum (n)	0.80-9.51 (21)	1.12-6.12 (8)	0.80-9.51 (13)
NLR (3-6 months)				
	Median [IQR]	2.14 [1.53-2.87]	2.44 [1.71-3.49]	1.87 [1.55-2.15]
	Minimum-Maximum (n)	1.09-7.04 (12)	1.26-7.04 (6)	1.09-5.06 (6)
NLR (6-12 months)				
	Median [IQR]	1.98 [1.76-3.07]	2.18 [2.18-2.18]	1.78 [1.74-3.76]
	Minimum-Maximum (n)	1.71-5.74 (4)	2.18-2.18 (1)	1.71-5.74 (3)
PLR (baseline)				
	Median [IQR]	162.42 [114.25-232.58]	133.45 [86.00-187.09]	168.62 [130.12-242.18]
	Minimum-Maximum (n)	72.22-305.70 (23)	72.22-274.21 (8)	101.55-305.70 (15)
PLR (1-3 months)				
	Median [IQR]	154.93 [112.75-215.66]	154.78 [112.26-216.95]	179.35 [136.63-215.66]
	Minimum-Maximum (n)	87.19-440.00 (21)	100.46-440.00 (8)	87.19-288.33 (13)
PLR (3-6 months)				
	Median [IQR]	181.23 [116.45-208.63]	194.58 [130.17-226.44]	148.49 [119.83-189.91]
	Minimum-Maximum (n)	75.23-252.16 (12)	75.23-237.74 (6)	96.88-252.16 (6)
PLR (6-12 months)				
	Median [IQR]	115.87 [79.01-144.18]	143.43 [143.43-143.43]	88.32 [69.70-117.37]
	Minimum-Maximum (n)	51.09-146.42 (4)	143.43-143.43 (1)	51.09-146.42 (3)

N: sample size; IQR: interquartile range; CgA: chromogranin A; GGT: gamma-glutamyltransferase; NLR: neutrophil-to-lymphocyte ratio; PLR: platelets-to-lymphocyte ratio.
